# Migraine and restless legs syndrome: is there an association?

**DOI:** 10.1007/s10194-011-0357-x

**Published:** 2011-06-10

**Authors:** Paul R. Cannon, Andrew J. Larner

**Affiliations:** Walton Centre for Neurology and Neurosurgery, Lower Lane, Fazakerley, Liverpool, L9 7LJ UK

**Keywords:** Migraine, Restless legs syndrome, Comorbidity, Dopamine, Sleep

## Abstract

Occasional clinical reports have suggested a link between migraine and restless legs syndrome. We undertook a systematic review of the evidence, which supports this association, and consider possible shared pathogenic mechanisms and the implications for current clinical practice.

## Introduction

Comorbidity may be defined as the association of two diseases in individuals at a frequency greater than that expected statistically by chance. Migraine may be complicated by the presence of a number of comorbidities (Table 1) [1, 2]. These comorbid conditions may augment the perceived severity of migrainous symptoms and/or pose additional challenges for effective patient management. It is therefore essential that any disease co-existing with migraine is identified and managed appropriately.

**Table 1:** Recognised comorbidities of migraine [1, 2]

Stroke
Subclinical vascular brain lesions
Coronary artery disease
Patent foramen ovale
Affective disorders: depression, anxiety
Epilepsy
Fibromyalgia
Possible comorbid conditions
Irritable bowel syndrome
Coeliac disease
Chronic fatigue syndrome
Raynaud’s phenomenon
Asthma
Narcolepsy
Sleep deprivation

Restless legs syndrome (RLS), also known as Ekbom’s syndrome [3], is a condition characterised by uncomfortable and sometimes painful sensory disturbances in the lower limbs producing an irresistible urge to move in order to relieve the sensation, particularly at rest and at night. RLS was not until recent times considered a possible migraine comorbidity [1]. Indeed, two recently published texts devoted to RLS reflect the uncertainty: Chaudhuri et al. [4] stated that RLS is seen in increased frequency in migraine cases, whereas Hening et al. [5] did not even mention migraine in their extensive (and contemporaneous) monograph.

Since RLS is a potentially treatable condition [4, 5], it is important to clarify whether it is comorbid with migraine. The availability of validated criteria for the diagnosis of RLS, the IRLSSG criteria [6], might facilitate study of this question. We therefore undertook a review of the available evidence which addresses this issue, and considered possible mechanisms of pathogenic overlap between migraine and RLS.

## Method

The medical databases Ovid, PubMed and NHS Evidence were searched by combining the key terms, migraine or migrain$ with restless legs syndrome. Electronic searches were supplemented by hand searching the bibliographies of relevant articles. Both authors read the title and abstract of all studies identified by the electronic searches, and agreed on eligibility of the studies identified, the full text of which was read and critically appraised.

## Results

From the articles retrieved (Table 2), we identified eight studies deemed eligible for inclusion [7–14].

**Table 2:** Articles retrieved by search strategy

Database	Results obtained
	Migraine or migrain$ and restless legs syndrome
Ovid (Medline)	12
PubMed	23
NHS evidence	50

No population-based study of the coincidence of migraine and RLS was identified, but several studies of clinical samples have been reported. Young et al. [7] screened 50 primary headache patients with intractable headache admitted to an outpatient infusion centre using the IRLSSG criteria. They reported that 34% of their patients met RLS criteria, higher than expected considering that the RLS prevalence rate in the general population has traditionally been stated to be between 1 and 10% [15, 16], although this may be lower in Asian populations [10]. Clearly, this was a small study sample and there was no control group, but the findings have been largely confirmed in subsequent studies.

Rhode et al. performed a case control study on 411 patients with migraine with 411 age- and sex-matched control subjects and found that the former had a statistically significant higher lifetime prevalence of RLS than the control group (17.3 vs. 5.6%, *p* < 0.001) [8]. It was also noted that migraine patients with RLS tended to be older than those without RLS, in keeping with the epidemiological finding that RLS incidence typically increases with age.

In an observational study, d’Onofrio et al. [9] conducted interviews and neurological examinations in 200 patients affected by primary headaches and in 120 age- and sex-matched controls. They reported an increased RLS prevalence in headache patients compared to control subjects (22.4 vs. 8.3%, *p* = 0.002), with a preponderance in patients who suffered from migraine without aura. Sleep disturbances were more frequent in headache patients with RLS (50.0 vs. 32.7%, *p* < 0.001).

Chen et al. [10] compared the prevalence of RLS between different primary headache groups. Headache diagnosis was based partly on physician interview and partly on patient-completed questionnaire, and RLS diagnosis was based on IRLSSG questionnaire confirmed by physician telephone interview. Of the 1,041 headache patients recruited, 772 had migraine, 218 tension-type headaches (TTH), and 51 cluster headaches (CH). There was no control group. The study found that RLS was more common in migraine patients (11.4%) than in TTH (4.6%) and CH (2.0%) (*p* = 0.002). The lower frequency of RLS in this population compared to the previously mentioned studies [7–9] suggested that an ethnic factor may contribute to RLS prevalence. Frequency of RLS increased with increasing number of migrainous symptoms (linear by linear association, *p* < 0.001).

Migraine patients with RLS had worse scores in six of the seven components of the Pittsburgh Sleep Quality Index (PSQI), a questionnaire used to assess sleep quality in the past month, in which total scores range from 0 to 21 with a score greater than 5 indicating poor sleep quality. Patients with migraine and RLS were found to be more likely to have a PSQI score greater than 5 compared to the patients with migraine alone (92.0 vs. 78.1%, *p* = 0.002). The study thus indicated that RLS might have a negative impact on sleep quality in migraine patients.

Limitations of these studies include the use of selected populations, use of patient questionnaires which may be subject to recall bias, and the adoption of cross sectional study designs which give only point, rather than lifetime, prevalence.

## Pathogenesis

Despite the potential limitations of these four clinical studies, they have consistently found that RLS is more common in migraine patients than in comparator groups. If migraine and RLS are comorbid, rather than simply coincident, are there shared aetiological factors and/or pathogenic mechanisms which might explain the association? A number of possible explanations have been offered for the association of RLS and primary headache, three of which (not mutually exclusive) have attracted most attention: dopaminergic dysfunction and dysfunctional brain iron metabolism; genetic linkage; and sleep disturbance.

### Dopaminergic dysfunction and dysfunctional brain iron metabolism

It has been postulated that RLS may be caused by dysfunction in central dopaminergic pathways. The strongest argument in favour of this is the rapid and dramatic improvement in RLS symptoms with dopamine agonist drugs which are now licensed for the treatment of RLS [17]. Animal models have shown that lesions to the A11 dopaminergic nucleus of the dorsal-posterior hypothalamus, the only nucleus providing dopaminergic stimulation to the spinal cord, produces a RLS-like phenotype [18].

Dopamine has also been implicated in migraine pathogenesis: there is evidence for dopamine receptor hypersensitivity in migraine patients [19]. Administration of low doses of dopamine agonists can trigger attacks of premonitory symptoms in migraineurs, including nausea, yawning and food craving. Conversely, dopamine antagonists may be of use in migraine treatment. Of note, when a trial of the dopamine antagonist droperidol was performed in acute migraine, a commonly reported side-effect was “acute drug-induced akathisia” [20], which might possibly have been RLS [21] since the two conditions may be difficult to differentiate clinically. Young et al. [7] also found that headache patients with RLS were at a greatly increased risk of developing drug-induced akathisia when treated with intravenous dopamine receptor blocking agents.

Cologno et al. [11] carried out a case control study on 164 patients with primary headache disorders screening each participant for the presence of RLS and premonitory migraine dopaminergic symptoms (yawning, nausea, somnolence or food craving). Concordant with previous studies, an increased incidence of RLS was found in migraineurs (25.6%). Intriguingly, those with co-existing migraine and RLS reported a higher prevalence of dopaminergic symptoms accompanying their migraine attacks than those without RLS (47.6 vs. 13.1%, *p* < 0.001), adding to the argument that a dopaminergic imbalance could be the pathogenic connection between migraine and RLS.

Iron dysmetabolism is well recognised in RLS, some cases being secondary to iron deficiency, and iron therapy being one approach to treatment of RLS [22]. There may be a link between brain iron and dopamine systems. Iron accumulation has also been documented in the brains of migraine patients [23].

### Genetic linkage

Individually, RLS and migraine seem to exhibit a polygenic inheritance pattern, both occurring with increased frequency in those with positive family histories. Currently, at least seven genetic loci have been linked to RLS [17, 24]. In addition, occasional pedigrees in which RLS and migraine appear to co-segregate over several generations have been presented [12, 13].

Sabayan et al. [14] suggested that it was credible to assume that migraine and RLS may have a joint origin, based in part on a common genetic linkage mapped to chromosome 14q21. This was described in an Italian family with idiopathic autosomal dominant RLS (OMIM %608831) [24]; one patient in the pedigree (III.17) had both RLS and migraine [25]. Soragna et al. [24] described a susceptibility locus for migraine without aura (OMIM %607501), also at 14q21, but there was no mention of RLS in affected individuals in this Italian family [26].

### Sleep disturbance

Sleep disorders are observed in a disproportionate number of patients with primary headache disorders [27]. Epidemiological studies indicate that sleep deprivation is one of the most common precipitating factors for migraine attacks [28, 29]. Improving sleep hygiene is often a component of headache management strategies, which alone may resolve or greatly improve headache complaints in some cases [30].

Given that RLS is, by definition, worse at nighttime, it is not surprising that the vast majority of RLS patients have sleep complaints [31]. Might the sleep disturbance caused by RLS exacerbate migraine and so explain the link between the two conditions? As previously mentioned, two of the epidemiological studies of migraine and RLS found sleep disturbance to be more evident in headache patients with RLS [9, 10].

Clinical trials have shown that treating RLS with dopamine agonists can enhance sleep quality [32]. However, there are currently no data which address the issue of whether treating RLS can improve the sleep of migraine patients and thus benefit migraine control, albeit that dopamine agonists may provoke premonitory migraine symptoms. Future studies might include a randomised control trial assessing this aspect of migraine management.

## Discussion

Studies in selected patient groups strongly suggest that RLS is more common in migraine patients than in control populations. However, a population-based study, perhaps the most definitive way to establish concurrence, has yet to be reported. It should be noted that certain conditions found to be comorbid with migraine in clinic-based studies were not confirmed as such in population-based studies (e.g. hypertension) [2].

There are a number of plausible hypotheses, which might explain a link between migraine and RLS. Of these, some have favoured dopaminergic imbalance. However, the opposing effects of dopaminergic agonists (improve RLS, provoke migraine premonitory symptoms) and antagonists (improve migraine, provoke drug-induced akathisia) in the two conditions would seem to negate any simplistic explanation at the level of neurotransmitter release or receptor sensitivity. Central mechanisms rendering the brain more sensitive to sensory stimuli with impaired habituation might be invoked, but precise mechanisms remain elusive. Hence, other explanations of the RLS-migraine link remain viable, of which sleep disturbance, as a consequence of RLS and as a trigger for migraine, remains an attractive possibility.

Whilst knowledge remains incomplete, what should be the pragmatic response to these data at the clinical interface? For example, should migraine patients attending the headache clinic be screened for RLS? Current (2010) guidelines from the British Association for the Study of Headache (www.bash.org.uk) do not mention RLS, far less advocate screening for it. It would certainly be difficult at this point in time to make a robust case for RLS screening in all headache patients, far less for measuring iron indices in all migraine patients. Nevertheless, it may be deemed good practice for clinicians to be vigilant for RLS symptoms in migraine patients, particularly in those with complaints of sleep disturbance or excessive daytime somnolence. Such screening for RLS might be performed with a single question [33]. However, even if a diagnosis of RLS is made, there is currently no evidence that specific RLS treatment will impact on migraine symptoms, indeed these drugs may cause adverse effects. Nonetheless, the possibility that improved sleep quality following treatment of RLS might be associated with improvement in migraine has some face validity.

Clearly more studies are required at both the basic and clinical level in this area to address the many outstanding questions. Trials in which both migraine and RLS symptoms are evaluated concurrently may help to inform clinical practice.
